# Experimental Senecavirus A Infection of Bovine Cell Lines and Colostrum-Deprived Calves

**DOI:** 10.3390/v14122809

**Published:** 2022-12-16

**Authors:** Alexandra Buckley, Lauren Crawford, Kyle Hoffman, Shollie Falkenberg

**Affiliations:** 1 Virus and Prion Research Unit, National Animal Disease Center, Agriculture Research Service, U.S. Department of Agriculture, Ames, IA 50010, USA; 2 Bacterial Diseases of Livestock Research Unit, National Animal Disease Center, Agriculture Research Service, U.S. Department of Agriculture, Ames, IA 50010, USA; 3 Ruminant Disease and Immunology Research Unit, National Animal Disease Center, Agriculture Research Service, U.S. Department of Agriculture, Ames, IA 50010, USA

**Keywords:** Senecavirus A, vesicular disease, cattle, susceptibility

## Abstract

Senecavirus A (SVA) is a causative agent for vesicular disease in swine, which is clinically indistinguishable from other vesicular diseases of swine including foot-and-mouth disease (FMD). Recently, it was reported that buffalo in Guangdong, China were experiencing clinical symptoms similar to FMD including mouth ulcers and lameness tested positive for SVA. The objective of this study was to determine the susceptibility of cattle (*Bos taurus*) to SVA infection. Initial in vitro work using the PrimeFlow assay demonstrated that bovine cell lines and peripheral blood mononuclear cells from cattle were susceptible to SVA infection. Subsequently, six colostrum-deprived Holstein calves were challenged with SVA intranasally. No vesicular lesions were observed after challenge. Serum, oral, nasal, and rectal swabs tested for SVA nucleic acid did not support significant viral replication and there was no evidence of seroconversion. Therefore, demonstrating cattle from this study were not susceptible to experimental SVA infection.

## 1. Introduction

Seneca Valley virus or Senecavirus A (SVA) was first discovered as a cell culture contaminant in 2002, and it was hypothesized that the virus could have been introduced via porcine trypsin or fetal bovine serum used commonly in cell culture work [1,2]. Subsequent serology testing suggested that both swine and cattle serum samples contained low neutralizing antibody titers against SVA [3]. Outbreaks of vesicular disease in swine across multiple countries occurred in 2015 with swine samples testing positive for SVA [4,5,6]. These findings were reproduced experimentally; thus, providing support for SVA being a causative agent for vesicular disease in swine and swine being the natural host [7,8,9]. 

However, recently there was a report of SVA isolation from an oral swab from a buffalo in Guangdong, China exhibiting clinical signs of mouth ulcers and lameness [10]. Since SVA causes clinical disease that is grossly indistinguishable to that caused by foot-and-mouth disease virus (FMDV), which infects cloven-footed animals and is a reportable disease to the World Organization of Animal Health, it is important to understand the potential host range of SVA [11]. Collectively, the reports describing low neutralizing antibody titers in bovine serum, and lesions in buffalo suggest susceptibility of bovines (subfamily Bovinae) which are comprised of cattle, bison, African buffalo, water buffalo, etc. Given the potential susceptibility of bovines, the objective of this study was to assess the susceptibility of cattle (*Bos taurus*) both in vitro and in vivo to experimental infection with SVA. 

## 2. Materials and Methods

### 2.1. Virus and Cell Culture

The SVA isolate utilized in all studies, NADC4 (MZ733977), was isolated on swine testicular (ST) cells from the lairage of a swine slaughter facility in 2020 and had been demonstrated to cause vesicular lesions in challenged pigs (Hoffman et al., unpublished). Cell lines utilized in this study included ST, porcine kidney (PK-15), Madin-Darby bovine kidney (MDBK), and bovine turbinate (BTu) cells. Cell lines were cultured in minimum essential medium (MEM) supplemented with L-glutamine (1.4 mM), 1% antibiotic-antimycotic, and 10% fetal bovine serum that was confirmed to be free of SVA and BVDV [12,13]. 

### 2.2. PBMC Isolation 

All study procedures were reviewed and approved by the Institutional Animal Care and Use Committee at the National Animal Disease Center (NADC) (ARS-2021-0982). Whole blood was collected from both swine and cattle in Vacutainer^®^ (BD) tubes containing acid citrus dextrose (ACD; Solution A). Peripheral blood mononuclear cells (PBMCs) were isolated using SepMate tubes (StemCell Technologies, Cambridge, MA, USA) according to manufacturer instructions and as previously described [14]. Next, 200 µL of each PBMC suspension containing 10^6^ cells were added to respective wells of a 96-well round bottom plate. Cells were plated in duplicate for both inoculated and non-inoculated wells, then incubated at 37 °C in a humid atmosphere of 5% CO_2_. After twenty-four hours, 50 µL of media was removed from the inoculation wells and replaced with 50 µL of NADC4 at an approximate MOI of 1. No media was changed in control wells. After 24 h, plates were centrifuged at 300× *g* for 4 min, and media was removed by flicking the plate. Cell pellets were washed and resuspended twice with 200 µL PBS, and pelleted by centrifugation at 300× *g* for 4 min. After the last wash step, the supernatant was discarded, and cell pellets were utilized for the PrimeFlow assay. 

### 2.3. Swine and Bovine Cell Lines

All cell lines were seeded to achieve approximately 80% confluency 24 h after seeding. Flasks (25 cm^2^) with each cell line (ST, PK-15, MDBK, and Btu) were inoculated at 24 h with NADC4 at an approximate MOI of 1. Non-inoculated flasks for each cell line were also maintained to evaluate non-specific binding of the SVA probe. 24 h post infection, cells were harvested from each flask and plated in a 96-well round bottom plate in the respective wells for further processing.

### 2.4. PrimeFlow Assay

All cell suspensions (cell lines and PBMCs) in the 96-well plate were centrifuged at 400× *g* for 4 min to pellet the cells. The cell pellets were washed with 200 µL of PBS to remove any residual medium and centrifuged again at 400× *g* for 4 min, twice. Cells in the 96-well plate were utilized in the PrimeFlow RNA assay, which was performed according to the manufacturer’s instructions. Proprietary specific oligonucleotide (RNA) probes targeting the VP1 protein of SVA were designed by Thermo Fisher Scientific in the type 1 channel (AF647). Flow cytometric analysis was performed using a BD FACSymphony^™^ A5 flow cytometer. Compensation beads from the PrimeFlow kit were used to determine the compensation settings. Frequency of cells positive for each respective cell line and target probe was calculated for each sample using FlowJo^®^ software (Tree Star, Inc., San Carlos, CA, USA).

### 2.5. Animal Study

Subsequently, ten colostrum-deprived (CD) female Holstein calves were purchased at approximately 6 weeks of age and delivered to the NADC in Ames, IA. Animals tested free of bovine viral diarrhea virus (BVDV) antibody and antigen as a measure of lack of exposure to viral pathogens during gestation. Immunologically naïve animals were selected as they have been used as a model for other viral susceptibility studies [15,16]. Upon arrival, the calves were assigned to the challenge (n = 6) or control (n = 4) group and separated into biosafety level 2 animal rooms in groups of 3 or 2, respectively. Animals were free of clinical lesions and baseline serum and swab (oral, nasal, and rectal) samples were collected during an approximate 1 week acclimation. A microchip was inserted in the neck region on the right side of each calf for temperature collection (Bio-Thermo^™^, Destron Fearing, TX, USA).

The inoculum was clarified and diluted in serum free MEM to a titer of 1 × 10^7^ TCID_50_/mL. The challenge group received 5 mL of inoculum intranasally on 0 days post inoculation (dpi). Animals were bled and oral, nasal, and rectal swabbed on 2–5, 8, 10, 12, and 15 dpi, and checked daily for clinical signs of vesicular disease (1–12 dpi). Clinical sign assessment included visualizing the coronary bands, soles of the feet, buccal and nasal mucus membranes, and the tongue for vesicles or erosions. Blood was also collected on 28 dpi. Blood samples were collected in serum separator and EDTA tubes. Swabs were placed in 1 mL of MEM. Whole blood was submitted for hematology analysis at the Iowa State Veterinary Diagnostic Lab the day of collection and serum and swab samples were stored at −80 °C for future testing. 

### 2.6. SVA Nucleic Acid Detection

SVA RNA extraction and real-time reverse transcriptase PCR testing was performed on serum and swab samples as previously described [13]. The primers and probe were designed to target nucleotides 602–710 of the genome. The forward primer sequence was 5′-TGCCTTGGATACTGCCTGATAG-3′, the reverse primer sequence was 5′-GGTGCCAGAGGCTGTATCG-3′, and the probe sequence was 5′-CGACGGCCTAGTCGGTCGGTT-3′. A dilution series was performed for a limit of detection (LOD) analysis and Ct values greater than 35 were considered negative. 

### 2.7. Virus Neutralization Assay

A virus neutralization (VN) assay was completed using heat inactivated serum as previously described [13]. Briefly, serum was serially diluted in MEM (1:4), incubated with an equal volume of NADC4 (~100 TCID_50_), and the solution was transferred to a confluent 96-well plate of ST cells. Titers were reported at the highest dilution of serum for which cytopathic effect was completely neutralized in ≥50% of the wells. 

## 3. Results

All swine and bovine cell lines and PBMCs were positive for SVA 24 h after inoculation as determined by flow cytometry using the PrimeFlow assay (Table 1). For MDBK, PK-15, and ST cell lines, 100% of the analyzed wells were positive for SVA. For BTu cells, 99.9% and 99.8% of cells in duplicate wells were positive for SVA (Table 1). The PBMCs from the four pigs and four calves had lower frequency of positive SVA cells. The average frequency of SVA positive swine PBMCs was 8.4% compared to 10.1% for bovine PBMCs (Table 1). The range for the frequency of SVA positive swine PBMCs was 5.2–12.3% compared to 7.3–13.8% for bovine PBMCs (Table 1). Due to the similarity in the susceptibility of both swine and bovine cell lines and PBMCs, in vivo research on the susceptibility of cattle to SVA infection began. 

After intranasal challenge of calves with SVA, no vesicular lesions were observed on the coronary bands, soles of the feet, buccal and nasal mucus membranes, and the tongue through 12 dpi. In addition, no animals developed lameness, and feed intake was consistent throughout the study. Diarrhea was noted in one room of challenged animals starting on 3 dpi until approximately 12 dpi. One challenged animal in the second room had an increased white blood cell count and increased lymphocytes starting on 4 dpi until approximately 8 dpi. No animals developed pyrexia during the challenge study. 

All serum and swab samples collected from control animals were PCR negative for SVA nucleic acid as well as most of the samples collected from challenged animals. Five samples tested PCR positive, and all C_t_ values were >33.9 (Table 2). The five PCR positive samples were spread among four of the challenged animals in nasal swabs or rectal swabs on 2 and 3 dpi. Baseline, 14 dpi, and 28 dpi sera samples were tested for SVA neutralizing antibodies, and all samples had titers of <1:4.

## 4. Discussion

Although the in vitro work showed that cattle could be susceptible to SVA infection, the in vivo results suggest there was no productive replication of SVA in CD calves after intranasal challenge. Colostrum-deprived calves were chosen due to their naïve immunologic status and have been used previously for viral susceptibility studies [15,16]. The PCR positive samples from nasal swabs from 2 and 3 dpi could be detection of residual challenge inoculum that was administered intranasally; however; minor replication of SVA in cattle cannot be ruled out, although, with most animals only having one PCR positive sample, it is less likely. 

This study did not find cattle to be susceptible to SVA infection; however, there are some differences between this study and the recent report of SVA isolation from a buffalo in Guandong, China [10]. The challenge isolate utilized in this study had a 96.8% nucleotide identity to the SVA strain the was isolated from the clinically affected buffalo in China (MN615881). Therefore, it is possible that differences between the strains could account for the lack of productive infection in this study. In addition, while both cattle and buffalo are in the subfamily Bovinae, cattle are from the Genus *Bos*, while buffalo are classified under *Bubalus,* which could have an impact on susceptibility. Differences between route of exposure and amount of virus can also not be compared due to the buffalo case occurring in the field and not in an experimental setting. 

Anthrax toxin receptor 1 (ANTXR1) was discovered to be the receptor for SVA in human cells, but has also been demonstrated to be the receptor in swine, as *ANTXR1* knockout pigs were resistant to SVA infection [17,18]. Previous in vitro work had shown that SVA can replicate in cattle cell lines, including MDBK cells [10]. Similarly, our work demonstrated the SVA can infect cattle cells lines and PBMCs. This work reinforces that in vitro cell culture susceptibility does not always reflect susceptibility of animals in vivo.

The World Organization for Animal Health lists FMDV as a notifiable disease and if it were to enter a naïve country, there would be severe economic ramifications [19]. Senecavirus A has become endemic in swine populations of multiple countries across the globe that also raise cattle. Since SVA causes vesicular lesions that are grossly identical to those caused by FMD, diagnostics must be performed to rule out FMDV, which could create a significant burden for the cattle industry, especially in FMDV negative countries. Based on this work, CD calves were not susceptible to experimental SVA infection; however, as new strains emerge, it is important to monitor for variants that may have a broader host range.

## Figures and Tables

**Table 1:** Frequency of Senecavirus A positive cells as determined by flow cytometry using the PrimeFlow assay.

Cell Type	Average	Minimum	Maximum
Bovine PBMC	10.1%	7.3%	13.8%
Porcine PBMC	8.4%	5.2%	12.3%
MDBK	100.0%	100.0%	100.0%
Btu	99.9%	99.8%	99.9%
PK-15	100.0%	100.0%	100.0%
ST	100.0%	100.0%	100.0%

**Table 2:** PCR C_t_ values of serum and swab samples after SVA challenge.

Cow #	Sample Type	Baseline	2 dpi	3 dpi	4 dpi	5 dpi	8 dpi	10 dpi	12 dpi
8927	Nasal swab	U	34.46	U	U	U	U	U	U
	Oral swab	U	U	U	U	U	U	U	U
	Rectal swab	U	U	U	U	U	U	U	U
	Serum	U	U	U	U	U	U	U	U
8929	Nasal swab	U	U	U	U	U	U	U	U
	Oral swab	U	U	U	U	U	U	U	U
	Rectal swab	U	34.54	U	U	U	U	U	U
	Serum	U	U	U	U	U	U	U	U
8938	Nasal swab	U	U	U	U	U	U	U	U
	Oral swab	U	U	U	U	U	U	U	U
	Rectal swab	U	U	U	U	U	U	U	U
	Serum	U	U	U	U	U	U	U	U
8931	Nasal swab	U	U	34.96	U	U	U	U	U
	Oral swab	U	U	U	U	U	U	U	U
	Rectal swab	U	34.18	U	U	U	U	U	U
	Serum	U	U	U	U	U	U	U	U
8944	Nasal swab	U	U	U	U	U	U	U	U
	Oral swab	U	U	U	U	U	U	U	U
	Rectal swab	U	U	U	U	U	U	U	U
	Serum	U	U	U	U	U	U	U	U
8945	Nasal swab	U	U	33.91	U	U	U	U	U
	Oral swab	U	U	U	U	U	U	U	U
	Rectal swab	U	U	U	U	U	U	U	U
	Serum	U	U	U	U	U	U	U	U

U = undetermined.

## Data Availability

The data presented in this study are available on request from the corresponding author.
